# A patient with CKD complicated by secondary hyperparathyroidism and parathyroid carcinoma: a case report

**DOI:** 10.3389/fmed.2026.1772235

**Published:** 2026-04-16

**Authors:** Wenxia Wang, Qiuchao Jin, Chengmin Huang

**Affiliations:** Changxing County People’s Hospital, Huzhou, Zhejiang, China

**Keywords:** case report, chronic kidney disease, malignancy, parathyroid carcinoma, secondary hyperparathyroidism

## Abstract

**Background:**

Parathyroid carcinoma (PC) is a rare malignancy, representing approximately 0.005% of all malignant tumors. Currently, its etiopathogenesis, diagnostic criteria, and optimal management strategies remain poorly defined. Furthermore, the occurrence of PC in the context of secondary hyperparathyroidism (SHPT) is an infrequent clinical event. This report details a case of PC arising in a patient with chronic kidney disease (CKD) and SHPT.

**Case presentation:**

A 53-year-old male with severe renal failure was admitted for clinical evaluation. During the course of hospitalization, SHPT and PC were diagnosed based on clinical, biochemical, and imaging evaluations. Intraoperative pathological assessment confirmed the malignancy, necessitating radical resection with regional lymph node dissection. The patient has achieved a long term disease free survival exceeding 8 years after surgery.

**Conclusion:**

The diagnosis of PC remains clinically challenging due to its rarity and the significant overlap in biochemical and imaging features with benign parathyroid lesions. This diagnostic challenge is further intensified in patients with severe renal insufficiency, as characteristic clinical indicators are often masked by comorbid secondary hyperparathyroidism. This report illustrates the diagnostic process and successful long-term management of PC in this challenging context, providing a clinical reference for the standardized treatment of this rare malignancy.

## Introduction

Parathyroid carcinoma (PC) is an exceptionally rare endocrine malignancy typically manifesting as hyperparathyroidism with significant renal and skeletal involvement (1). In contrast, secondary hyperparathyroidism (SHPT) is a prevalent complication of chronic kidney disease (CKD) that exacerbates with the progression of renal dysfunction. The impaired renal function leads to phosphate retention, which stimulates fibroblast growth factor 23 (FGF23) secretion and reduces active vitamin D levels (2, 3). These metabolic alterations drive parathyroid hyperplasia and excessive parathyroid hormone (PTH) secretion, resulting in the development of SHPT (4).

The pathophysiological mechanisms linking CKD to PC remain an area of ongoing research. CKD, particularly in its advanced stages, leads to significant metabolic disturbances such as altered calcium-phosphate balance, dysregulation of FGF23, and reduced vitamin D activation (5). These factors contribute to parathyroid hyperplasia, which may, in rare cases, progress to malignancy. The exact mechanisms are not fully understood, but the chronic state of hyperparathyroidism in CKD patients likely plays a significant role in the development of parathyroid carcinoma (2, 6).

The diagnostic ambiguity of PC is significantly amplified when it co-exists with SHPT, as the clinical and biochemical hallmarks of malignancy, such as extreme parathyroid hormone (PTH) elevation and profound hypercalcemia, which are often indistinguishable from the manifestations of severe renal-induced hyperparathyroidism (7, 8). This diagnostic overlap frequently leads to suboptimal initial surgical interventions. Specifically, the failure to perform a radical comprehensive radical resection during the primary operation substantially increases the susceptibility to local recurrence and distant metastasis (9). Furthermore, delayed or inadequate management of PC in this context results in a poor prognosis, as patients are predisposed to refractory and life-threatening hypercalcemia (9). As PC is typically characterized as an indolent tumor, mortality is primarily driven by these severe metabolic derangements rather than the direct physiological effects of the tumor burden (10).

This report describes a case of concurrent PC and SHPT with over 8 years of disease-free survival. Documentation of this clinical course provides a reference for the diagnosis and surgical management of this rare malignancy in patients with renal failure.

## Case report

In 2010, a 47-year-old male was found to have a serum creatinine level of approximately 200 μmol/L during a routine physical examination. Although the patient presented with persistent foamy urine, the absence of acute discomfort led to a period of clinical observation without formal intervention. In October 2017, the patient underwent surgery for a fracture at an external hospital, during which clinical evaluations confirmed that the kidney function had progressed to end-stage renal disease, necessitating the initiation of temporary hemodialysis. On November 22, 2017, the patient was admitted for further management of uremia and initiated peritoneal dialysis. Laboratory investigations upon admission revealed severe anemia with a hemoglobin level of 66 g/L and profound renal dysfunction, indicated by a serum creatinine of 861.1 μmol/L and urea nitrogen of 14.1 mmol/L (Table 1). The PTH level of 1,925 ng/L, serum calcium of 2.73 mmol/L, serum phosphorus of 1.98 mmol/L, and an elevated alkaline phosphatase level of 322 U/L collectively indicated significant SHTP (Table 1). Thyroid function tests showed a thyroglobulin antibody level of 433 IU/mL and a free thyroxine level of 0.56 ng/dL. Physical examination upon admission identified a palpable mass on the right side of the neck measuring approximately 4.0 by 4.0 centimeters. The mass exhibited clear boundaries but limited mobility, and no tremors or vascular murmurs were detected upon auscultation. Cardiovascular and abdominal examinations revealed no significant abnormalities.

**Table 1:** Blood test results.

Blood test	Result	Reference value	Unit
White blood cell	5,000	3,500–9,500	/mm^3^
Hemoglobin	66	115–150	g/L
Platelet count	18.5	12.5–35.0	10^4^/μL
Total protein	67.8	65–85	g/L
Albumin	42.9	40–55	g/L
Urea nitrogen	14.1	2.5–7.5	mmoL/L
Creatinine	861.1	30–130	umoL/L
Sodium	144.2	137–147	mmoL/L
Potassium	3.8	3.5–5.3	mmoL/L
Chloride	109.7	99–110	mmoL/L
Calcium	2.73	2–2.7	mmoL/L
Phosphorus	1.98	0.81–1.55	mmoL/L
Asparate aminotransferase	11	15–40	IU/L
Alanine aminotransferase	11	9–50	IU/L
Alkaline phosphatase	322	50–135	U/L
Lactate dehydrogenase	129	80–240	IU/L
Total bilirubin	5.8	5.1–26	mmoL/L
Thyroid-Stimulating Hormone	0.81	0.27–4.2	IU/mL
Free Triiodo thyronine	2.98	2–4.4	pg/mL
Free Thyroxine	0.56	0.93–1.7	ng/dL
Thyroglobulin antibody	433	<115	IU/mL
Intact parathyroid hormone (PTH)	1925	15–65	ng/L

**Figure 1:**
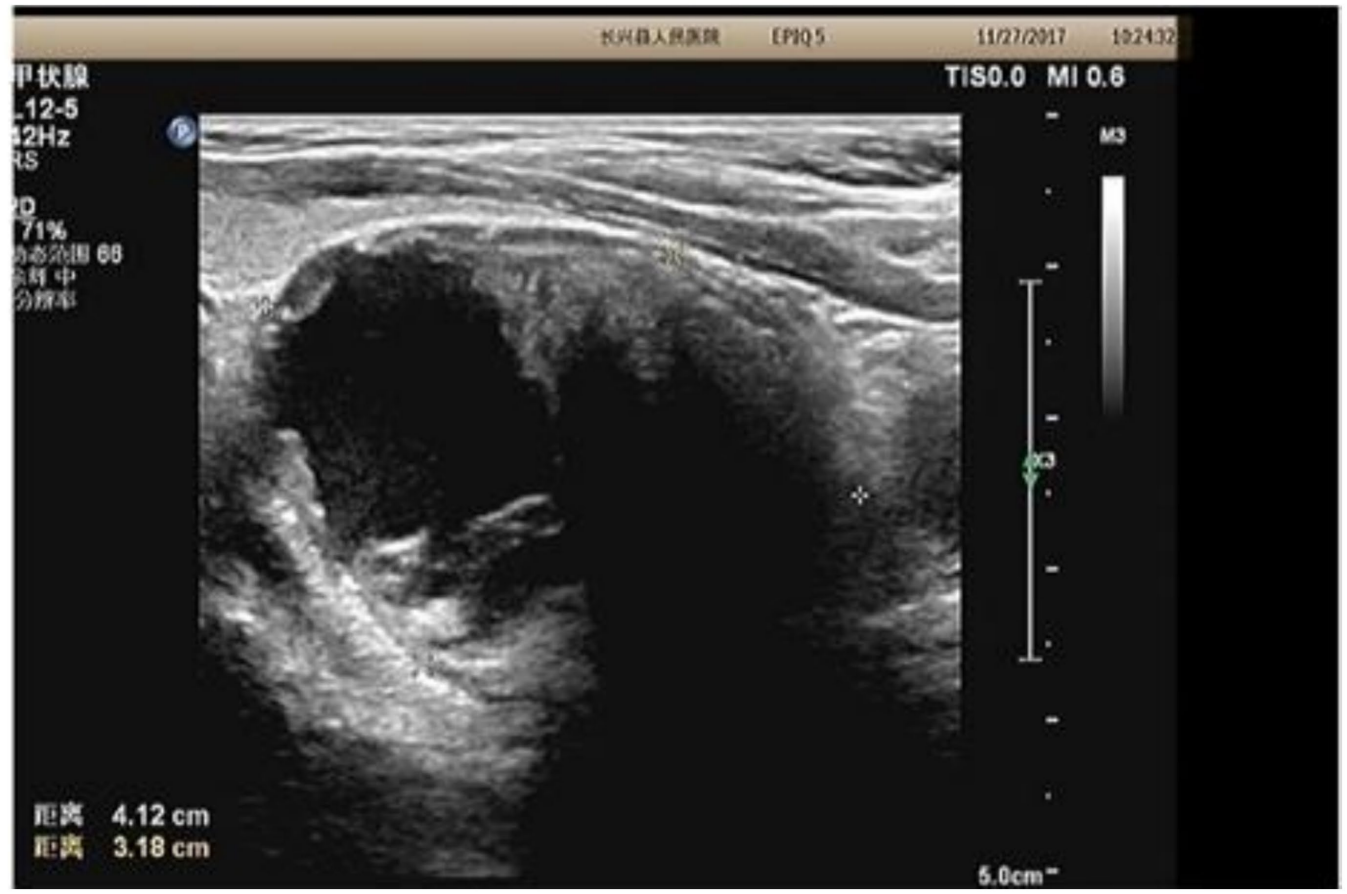
Preoperative ultrasound of the thyroid gland thyroid (ultrasound)|parathyroid findings were: multiple cystic solid nodules of the thyroid gland (TI-RADS category 3), cystic solid mass posterior to the inferior pole of the thyroid gland in the right lobe with calcification (cystic solid echogenicity of 41 × 32 mm), and parathyroid adenoma with hemorrhagic cystic degeneration. Parathyroid (ultrasound) findings were: cystic solid mass with calcification posterior to the inferior pole of the thyroid gland in the right lobe, and parathyroid adenoma with hemorrhagic cystic degeneration.

**Figure 2:**
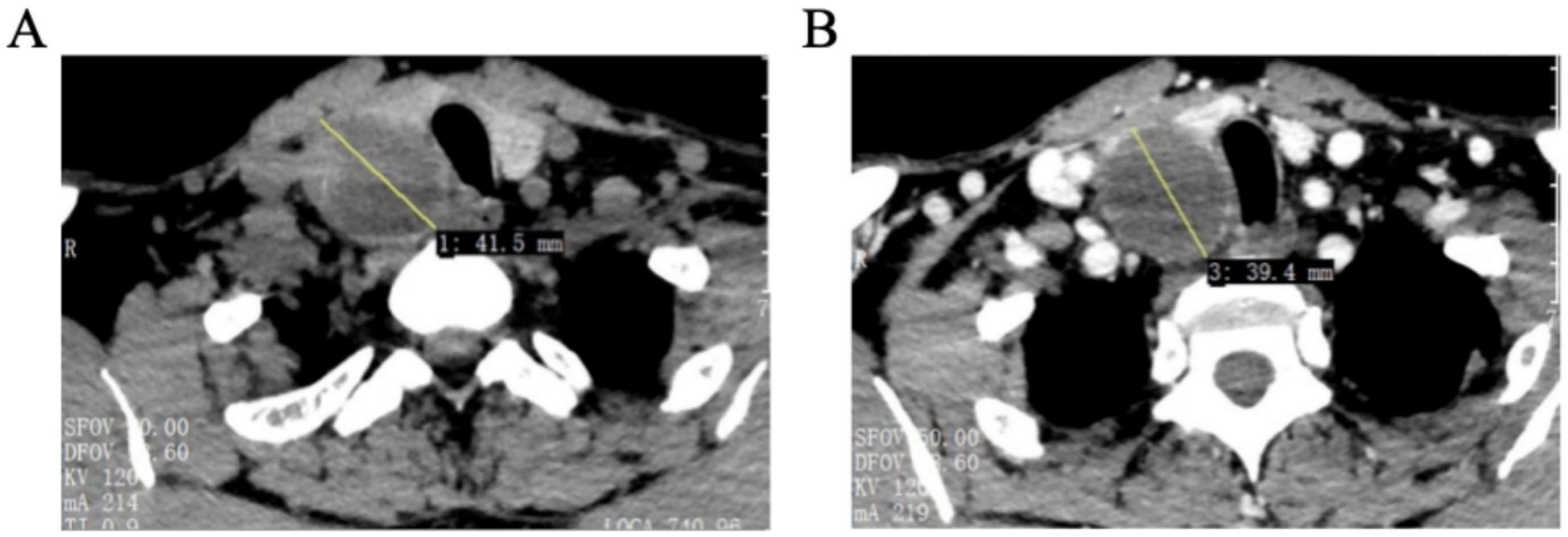
**(A)** Plain scan CT and **(B)** enhanced CT. Cervical plain plus contrast-enhanced CT findings were: 1. Hypodense nodular shadow of the left thyroid gland; 2. Oval cystic solid mass shadow of the right inferior thyroid gland, and parathyroid adenoma with partial cystic degeneration after first examination (please combine with other related examinations).

To further evaluate the palpable cervical mass and investigate the underlying cause of the abnormal thyroglobulin antibody and free thyroxine levels, diagnostic imaging was performed. Thyroid and parathyroid ultrasonography identified multiple cystic-solid nodules in the thyroid gland classified as TI-RADS category 3 (Figure 1). A cystic solid mass measuring 41 by 32 millimeters with internal calcification was located posterior to the inferior pole of the right thyroid lobe (Figure 1). Cervical plain plus contrast-enhanced CT confirmed the presence of an oval cystic solid mass in the right inferior thyroid region (Figure 2). Based on these findings and the context of secondary hyperparathyroidism, the preoperative diagnosis was established as a right inferior parathyroid tumor, initially suspected to be a parathyroid adenoma with potential hemorrhagic cystic degeneration. Surgical intervention was indicated to address the suspected parathyroid tumor.

On December 16, 2017, the patient was referred to the Department of Thyroid and Breast Surgery for surgery. During the procedure, an enlarged right inferior parathyroid gland was identified with dimensions of 3.5 by 4.5 centimeters. In contrast, the contralateral parathyroid glands measured between 0.8 and 1.2 centimeters, indicating asymmetric glandular involvement. The right inferior parathyroid gland was excised and submitted for intraoperative frozen section analysis. The pathological assessment revealed an epithelial tumor characterized by a vesicular pattern and invasive growth into the interstitial fibrous tissue (Supplementary material 1). Given the high clinical suspicion of malignancy based on these intraoperative findings, the surgical plan was immediately modified from a simple excision to a comprehensive radical resection. This procedure included a right inferior parathyroidectomy, a right thyroid lobectomy, and a right central lymph node dissection.

The definitive postoperative pathological report provided the basis for the final diagnosis of parathyroid carcinoma (Supplementary material 2). The parathyroid tumor measured 5.0 by 4.5 by 3.5 centimeters and contained melanin pigmentation along with degenerative and cystic changes. Microscopic examination confirmed that the carcinoma had invaded the fibrous capsule and reached the peritumoral vasculature, with vascular invasion identified in more than five distinct locations. All nine regional lymph nodes recovered from the right central zone were negative for malignancy. Further immunohistochemical analysis supported this diagnosis (Supplementary material 3), as the tumor cells demonstrated strong membrane expression of beta-catenin and a Ki-67 proliferation index of 1 percent. Crucially, the cells were negative for thyroid transcription factor 1 (TTF-1) and thyroglobulin (TG), which excluded thyroid follicular epithelial differentiation and confirmed the parathyroid origin of the malignancy. Additional special staining with periodic acid-Schiff (PAS) confirmed the presence of glycogen in the cytoplasm, further supporting the diagnosis of parathyroid carcinoma with extensive vascular invasion.

**Figure 3:**
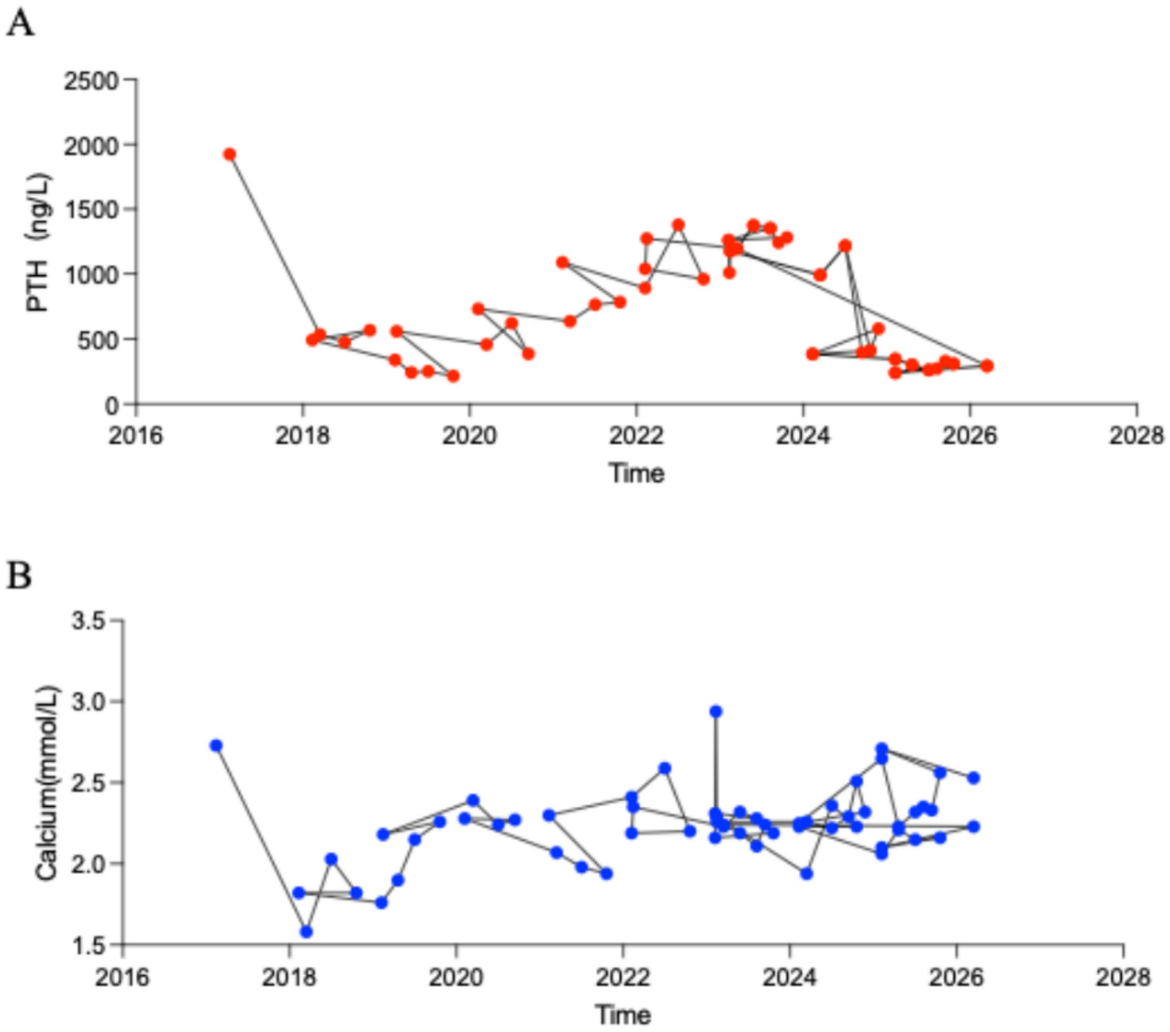
Line graph of changes in blood PTH and blood calcium. **(A)** The blood PTH change curve during hospitalization and follow-up in our hospital. **(B)** The blood calcium change curve during hospitalization and follow-up in our hospital.

Following the surgical intervention, the patient was managed with 0.5 μg softgel capsule for oral QD and 50 μ salmon calcitonin injection for intramuscular QD. Long-term management included continuous hemodialysis and regular clinical surveillance. Throughout the follow-up period, the patient remained clinically stable with no evidence of tumor recurrence. By November 2021, follow-up evaluations showed that the PTH level had risen to approximately 1,000 ng/mL (Figure 3A), and thyroid ultrasound suggested potential hyperplasia of the left parathyroid gland (Supplementary material 4). While these findings indicated a recurrence of secondary hyperparathyroidism, the serum calcium level remained within the normal range (Figure 3B). As of the latest assessment, the patient remains alive with no clinical or radiological signs of parathyroid carcinoma recurrence, achieving a disease-free survival exceeding 8 years post-intervention.

## Discussion

PC is an exceptionally rare endocrine malignancy, representing approximately 0.005% of all malignant tumors and 0.3 to 5.6% of cases involving primary hyperparathyroidism (11, 12). According to the 2022 World Health Organization (WHO) classification of parathyroid tumors, the histological diagnosis of PC remains contingent upon clear evidence of invasive growth (13). These criteria include vascular invasion, lymphatic invasion, perineural or intraneural invasion, and local malignant infiltration into adjacent anatomical structures. Despite these established pathological benchmarks, the clinical and biochemical identification of PC remains challenging, particularly when the malignancy occurs concurrently with SHPT in the setting of CKD.

In patients with CKD, the metabolic disturbances associated with renal failure significantly complicate the diagnostic reasoning for PC (14). Pathophysiologically, impaired renal function leads to phosphate retention, reduced active vitamin D levels, and elevated fibroblast growth factor 23 (FGF23), all of which drive parathyroid hyperplasia and excessive PTH secretion (15, 16). A critical diagnostic pitfall in this context is the masking effect of CKD on the biochemical presentation of parathyroid malignancy. While PC typically manifests with profound hypercalcemia, the systemic alterations of renal failure can suppress serum calcium levels, resulting in only mild elevations despite markedly increased PTH concentrations (17). Consequently, the characteristic biochemical hallmarks of PC may be indistinguishable from those of severe SHPT, necessitating a heightened reliance on meticulous histopathological evaluation and intraoperative findings to distinguish PC from benign parathyroid lesions or atypical tumors (18, 19).

The differentiation among parathyroid adenoma, atypical parathyroid tumors, and PC relies on specific architectural hallmarks rather than cellular atypia alone (13). According to current histopathological criteria, parathyroid adenomas are benign neoplasms lacking any evidence of invasive growth. Atypical parathyroid tumors occupy an intermediate category, exhibiting worrisome features such as dense fibrous bands, focal necrosis, or increased mitotic activity, yet they lack unequivocal evidence of capsular or vascular penetration. In the present case, the diagnosis of PC was established based on the identification of definitive vascular invasion in more than five distinct locations and the infiltration of the tumor cells into the surrounding fibrous capsule. These findings fulfill the essential requirements for malignancy, distinguishing the lesion from both benign adenomas and atypical parathyroid neoplasms.

Regarding the immunohistochemical profile, a Ki-67 proliferation index of 1 percent was recorded in this case. Although a low Ki-67 proliferation index is unusual for many malignant tumors, it does not exclude a diagnosis of PC when unequivocal histological evidence of invasion is present (13, 18). In parathyroid malignancies, the diagnostic significance of the proliferation index is often secondary to the objective observation of angioinvasion or lymphatic spread (13). Additionally, the negative expression of thyroid-specific markers, such as TTF 1 and TG, confirmed the parathyroid origin of the tumor and excluded the possibility of metastatic thyroid follicular carcinoma. Furthermore, the rare identification of melanin pigmentation and the presence of cytoplasmic glycogen, as confirmed by PAS, highlight the unique pathological characteristics of this malignancy and provide additional evidence of its parathyroid lineage.

Reports of PC in patients with end-stage renal disease are infrequent, and many documented cases note a potential for recurrence (9, 14, 20). This case presents a disease-free survival period of 8 years, which serves as a relatively long-term follow-up record for this rare clinical scenario. The combination of these clinical findings and the management of associated metabolic disturbances provides a reference for the documentation of parathyroid malignancy in patients with renal failure.

The choice of surgical approach is a key consideration in the management of PC (6, 9). While parathyroidectomy is the standard treatment for secondary hyperparathyroidism, the identification of asymmetric enlargement or suspicious features during exploration may indicate a transition to a radical resection. In this instance, the decision to perform a right thyroid lobectomy and central lymph node dissection, rather than a simple tumor excision, appeared to be beneficial in preventing local recurrence. This illustrates the role of intraoperative assessment, particularly when preoperative biochemical markers are influenced by the masking effect of renal failure.

In summary, the coexistence of PC and secondary hyperparathyroidism poses a diagnostic challenge. Maintaining clinical vigilance when encountering large or invasive parathyroid lesions in patients with renal failure is appropriate, even when serum calcium levels are only mildly elevated. A radical resection combined with consistent follow-up represents a management strategy for achieving stable clinical outcomes in this patient population.

## Conclusion

We report a case of a patient with CKD complicated by SHPT and PC. Surgery was the only treatment option for this condition, and the patient has now achieved an 8-year survival period without tumor recurrence.

## Data Availability

The raw data supporting the conclusions of this article will be made available by the authors, without undue reservation.
