# Supplementary material for: A patient with CKD complicated by secondary hyperparathyroidism and parathyroid carcinoma: a case report
Source: Front Med (Lausanne). 2026 Apr 16;13:1772235. doi: 10.3389/fmed.2026.1772235 (PMC13128398; doi:10.3389/fmed.2026.1772235)
Supplement: Supplementary file 1 [file Data_Sheet_1.pdf]

长兴县人民医院

Zhejiang University Second Affiliated Hospital, Remote Medical Center

浙医二院远程医学中心

病理咨询报告单

Pathology Consultation Report

Pathology No.: F17-0762

病理号：F17-0762

送检日期：2017-12-16

Specimen Receipt Date: 2017-12-16

[redacted]

送检材料：右侧甲状腺　Specimen Submitted: Right Thyroid

附图： Attached Figures:

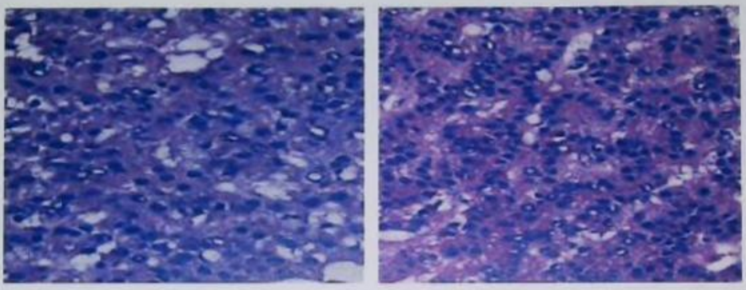

会诊意见： Consultation Opinion:

(右侧甲状旁腺？)上皮性肿瘤，呈腺泡状，侵袭性生长，伴间质纤维组织增生。结合临床，考虑甲状旁腺腺瘤，囊性变，部分癌变。

(Right parathyroid?) Epithelial tumor with acinar pattern, showing invasive growth and accompanied by stromal fibrosis. Combined with clinical findings, it is considered to be a parathyroid adenoma with cystic change and partial carcinomatous transformation.
