# Supplementary material for: A patient with CKD complicated by secondary hyperparathyroidism and parathyroid carcinoma: a case report
Source: Front Med (Lausanne). 2026 Apr 16;13:1772235. doi: 10.3389/fmed.2026.1772235 (PMC13128398; doi:10.3389/fmed.2026.1772235)
Supplement: Supplementary file 2 [file Data_Sheet_2.pdf]

Changxing County People's Hospital Pathology

长兴县人民医院病理

病理诊断报告单

Pathology Diagnostic Report

Pathology No.: 2017-21297

病检号: 2017-21297

[redacted]

收到日期: 2017-12-16

Receipt Date: 2017-12-16

送检材料: 右甲状腺组织

临床诊断: 右甲状腺待查

Specimen Submitted: Right Thyroid Tissue

Clinical Diagnosis: Right Thyroid Under Investigation

附图:

Attached Figures:

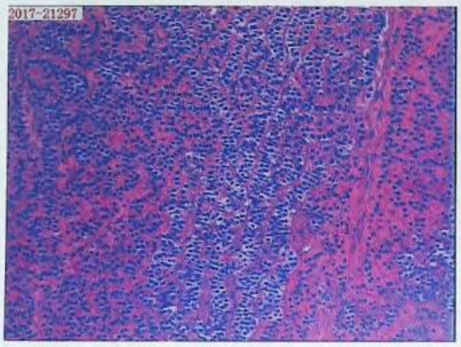

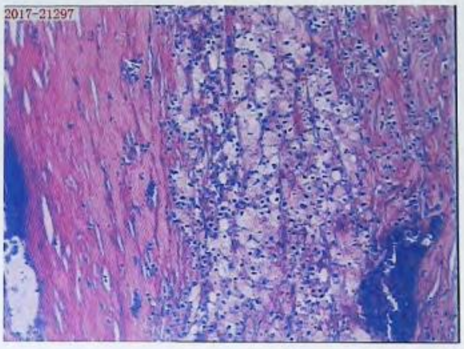

Gross Examination and Pathological Diagnosis:

巨检及病理诊断:

(右下甲状旁腺) 甲状旁腺腺瘤，部分肿瘤细胞含有黑色素，伴瘤组织退行性变和囊性变。囊壁纤维化和钙化。部分癌变，侵犯纤维包膜和包膜血管。包膜血管侵犯>5处。大小5cm×4.5 cm×3.5 cm。未见穿透纤维包膜。
(右中央区上淋巴结)纤维脂肪组织。
（右中央区下淋巴结）淋巴结0/9阳性。
（右侧甲状腺叶）结节性甲状腺肿。
（右侧中央区上淋巴结2）纤维血管脂肪组织。
（喉前淋巴结）甲状腺组织和横纹肌组织。
建议临床，进一步免疫组化检查和特殊染色。
补充特殊染色，PAS染色示肿瘤细胞胞质含有糖原。弹力纤维染色见于小动脉壁，未见肿瘤累及动脉。

免疫组化
R片: CD10(-)、CD117(-)、CK-H(+)、Ki-67(1%)、HMB45(-)、melan-A(-)、syn(±)、TTF-1(-)、Tg(-)、β-catenin(+++，膜)、P53(-)、CD34(+，血管内皮)。提示肿瘤细胞不表达甲状腺滤泡上皮分化的标志物如TTF-1和TG。符合甲状旁腺腺瘤，部分癌变，侵犯包膜血管阳性

(Right Inferior Parathyroid) Parathyroid adenoma. Partial tumor cells contain melanin pigment, accompanied by degenerative changes and cystic changes in the tumor tissue. Cyst wall fibrosis and calcification are present. Partial carcinomatous transformation, with invasion of the fibrous capsule and capsular blood vessels. Capsular vascular invasion >5 sites. Size: 5 cm × 4.5 cm × 3.5 cm. No penetration of the fibrous capsule is observed.

(Right Central Superior Lymph Node) Fibroadipose tissue.

(Right Central Inferior Lymph Node) Lymph nodes 0/9 positive.

(Right Thyroid Lobe) Nodular goiter.

(Right Central Superior Lymph Node 2) Fibrovascular adipose tissue.

(Pretracheal Lymph Node) Thyroid tissue and skeletal muscle tissue.

Recommendation for Clinical Management: Further immunohistochemical (IHC) studies and

special staining.

Supplementary Special Staining: PAS staining demonstrates glycogen in the cytoplasm of tumor cells. Elastic fiber staining is observed in the walls of small arteries, with no tumor involvement of the arteries.

Immunohistochemistry (IHC):

R: CD10(-), CD117(-), CK-H(+), Ki-67(1%), HMB45(-), Melan-A(-), Syn(±), TTF-1(-), Tg(-), β-catenin(+++, membranous), P53(-), CD34(+, vascular endothelium).

Interpretation: The tumor cells do not express markers of thyroid follicular epithelial differentiation, such as TTF-1 and Tg. Findings are consistent with a parathyroid adenoma, partially carcinomatous, with positive capsular vascular invasion.
