# Supplementary material for: A patient with CKD complicated by secondary hyperparathyroidism and parathyroid carcinoma: a case report
Source: Front Med (Lausanne). 2026 Apr 16;13:1772235. doi: 10.3389/fmed.2026.1772235 (PMC13128398; doi:10.3389/fmed.2026.1772235)
Supplement: Supplementary file 3 [file Data_Sheet_3.pdf]

Changxing County People's Hospital Pathology

长兴县人民医院病理

免疫组化报告单

Immunohistochemistry Report

病理号：2017-21297 免疫组化号：I2017-0598

Pathology No.：2017-21297 [redacted] Immunohistochemistry No.：2017-0598

[redacted]

Date of Report：2017-12-31

[redacted] 诊断日期:2017-12-31

送检标本：右甲状腺组织 Specimen Submitted：Right Thyroid Tissue

[redacted]

附图： Attached Figures：

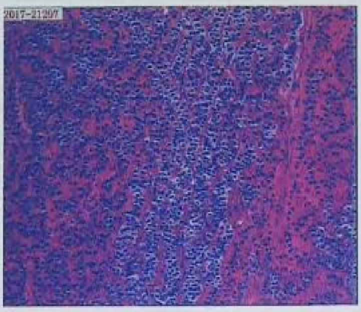

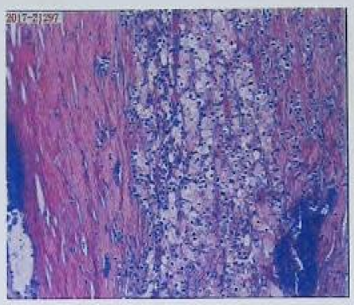

免疫组化结果： Immunohistochemistry Results：

R片：CD10(-)、CD117(-)、CK-H(+)、Ki-67(1%)、HMB45(-)、melan-A(-)、syn (±)、TTF-1(-)、Tg(-)、β-catenin(+++，膜)、P53(-)、CD34(+，血管内皮)。提示肿瘤细胞不表达甲状腺滤泡上皮分化的标志物如TTF-1和TG。符合甲状旁腺腺瘤，部分癌变，侵犯包膜血管阳性。

R: CD10(-), CD117(-), CK-H(+), Ki-67(1%), HMB45(-), Melan-A(-), Syn(±), TTF-1(-), Tg(-), β-catenin(+++, membranous), P53(-), CD34(+, vascular endothelium).

Interpretation: The tumor cells do not express markers of thyroid follicular epithelial differentiation, such as TTF-1 and Tg. Findings are consistent with a parathyroid adenoma, partially carcinomatous, with positive capsular vascular invasion.
