# Supplementary material for: A patient with CKD complicated by secondary hyperparathyroidism and parathyroid carcinoma: a case report
Source: Front Med (Lausanne). 2026 Apr 16;13:1772235. doi: 10.3389/fmed.2026.1772235 (PMC13128398; doi:10.3389/fmed.2026.1772235)
Supplement: Supplementary file 4 [file Data_Sheet_4.pdf]

Changxing County People's Hospital (Zhejiang University Second Affiliated Hospital Changxing Branch)

长兴县人民医院(浙医二院长兴院区)

超声检查报告单

Ultrasonography Report

仪器名称：EPIQ-7C(Philips)

Examined Area: Parathyroid Glands (Ultrasound)

Examination No.: US2048000 Instrument: EPIQ-7C (Philips)

检查部位：甲状旁腺（B超） 检查号：US2048000 检查日期：2022-10-26 Examination Date: 2022-10-26

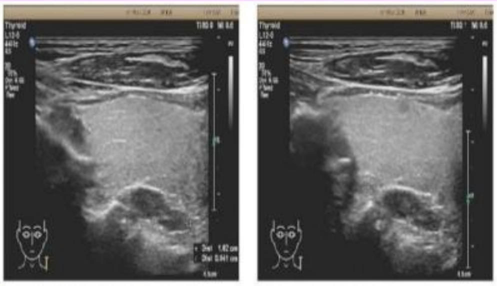

超声描述：左侧甲状腺背侧后方可见大小约1.8*0.6cm的低回声影，右侧甲状旁腺区扫查目前未见异常包块回声。

Ultrasound Findings:

A hypoechoic lesion measuring approximately 1.8 x 0.6 cm is noted posterior to the dorsal aspect of the left thyroid lobe. No abnormal mass echo is currently detected in the right parathyroid region.

Ultrasound Diagnosis:

A hypoechoic lesion posterior to the dorsal aspect of the left thyroid lobe, suggestive of possible parathyroid hyperplasia. Clinical correlation is recommended.

超声诊断：左侧甲状腺背侧后方低回声影，甲状旁腺增生可能，请结合临床
